# Analysis of structural variation among inbred mouse strains

**DOI:** 10.1186/s12864-023-09197-5

**Published:** 2023-03-02

**Authors:** Ahmed Arslan, Zhuoqing Fang, Meiyue Wang, Yalun Tan, Zhuanfen Cheng, Xinyu Chen, Yuan Guan, Laura J. Pisani, Boyoung Yoo, Gill Bejerano, Gary Peltz

**Affiliations:** 1 Department of Anesthesia, Pain and Perioperative Medicine, Stanford University School of Medicine, 94305 Stanford, CA USA; 2 Department of Radiology, 94305 Stanford, CA USA; 3 Dept. of Computer Science, Stanford School of Engineering, 94305 Stanford, CA USA; 4 Developmental Biology, Biomedical Data Science, Stanford School of Medicine, 94305 Stanford, CA USA

**Keywords:** Genetic analysis, Mouse genetic models, Structural variation

## Abstract

**Background:**

‘*Long read*’ sequencing methods have been used to identify previously uncharacterized structural variants that cause human genetic diseases. Therefore, we investigated whether long read sequencing could facilitate genetic analysis of murine models for human diseases.

**Results:**

The genomes of six inbred strains (BTBR T + Itpr3tf/J, 129Sv1/J, C57BL/6/J, Balb/c/J, A/J, SJL/J) were analyzed using long read sequencing. Our results revealed that (i) Structural variants are very abundant within the genome of inbred strains (4.8 per gene) and (ii) that we cannot accurately infer whether structural variants are present using conventional short read genomic sequence data, even when nearby SNP alleles are known. The advantage of having a more complete map was demonstrated by analyzing the genomic sequence of BTBR mice. Based upon this analysis, knockin mice were generated and used to characterize a BTBR-unique 8-bp deletion within *Draxin* that contributes to the BTBR neuroanatomic abnormalities, which resemble human autism spectrum disorder.

**Conclusion:**

A more complete map of the pattern of genetic variation among inbred strains, which is produced by long read genomic sequencing of the genomes of additional inbred strains, could facilitate genetic discovery when murine models of human diseases are analyzed.

**Supplementary Information:**

The online version contains supplementary material available at 10.1186/s12864-023-09197-5.

## Background

While commonly used next generation sequencing methods analyze ~ 200–300 bp DNA segments (i.e., ‘*short read*’ (**SR**) sequencing) [1], recently developed ‘*long read*’ (**LR**) sequencing methods that can analyze 20 kb DNA segments [2, 3] have enabled previously uncharacterized structural variants (**SV**) (i.e., genomic alterations > 50 bp in size) to be evaluated. LR sequencing has been used to characterize genetic disease mechanisms that could not otherwise be analyzed [1–3]; which include large genomic alterations in patients with Cardiac Myxomata (*PRKAR1A*) [4], Bardet–Biedl syndrome (*BBS9)* [5] and intellectual disability (*ARHGEF9*) [6]. Mouse is the premier model organism for biomedical discovery, and many genetic factors affecting important biomedical traits have been identified by analyzing mouse genetic models [7, 8]. However, prior analyses of SNP [9, 10] and SV [11–13] alleles among inbred strains utilized SR genomic sequence, which has a limited ability to fully characterize SV. As with human diseases, a more complete map of the pattern of genetic variation, which accurately catalogues SVs among inbred mouse strains, could enable genetic discovery.

Therefore, LR sequencing was utilized to evaluate SVs in six inbred mouse strains. Five of these are commonly used inbred strains that exhibit outlier phenotypes for important biomedical traits that include resistance to acetaminophen-induced liver toxicity (SJL) [14], susceptibility to haloperidol-induced CNS toxicity (A/J) [15], and resistance to developing opiate dependence (129Sv1) [16]. Another strain (BTBR T + Itpr3tf/J, **BTBR**) uniquely displays neuroanatomic abnormalities and behaviors that are characteristic of human Autism Spectral Disorder (**ASD**) [17–20]: (i) the neuroanatomic changes include a complete absence of a corpus callosum (**CC**); (ii) a deficiency in engaging in social tasks; and (iii) abnormal repetitive behaviors [20, 21]. Despite the multiple studies performed to date - which have used epigenetic [22], genetic [23], transcriptomic [24–26] and proteomic [24, 27] methodologies - the genetic basis for the BTBR abnormalities is not known. When the LR genomic sequence for six strains was analyzed along with SR sequence for 53 strains, we found that SVs are abundant in the genome of inbred mouse strains. Therefore, we investigated whether having a more complete map of the pattern of genetic variation in the BTBR genome could facilitate identification of a genetic factor that contributes to its ASD-like abnormalities.

## Results

**Fig. 1:**
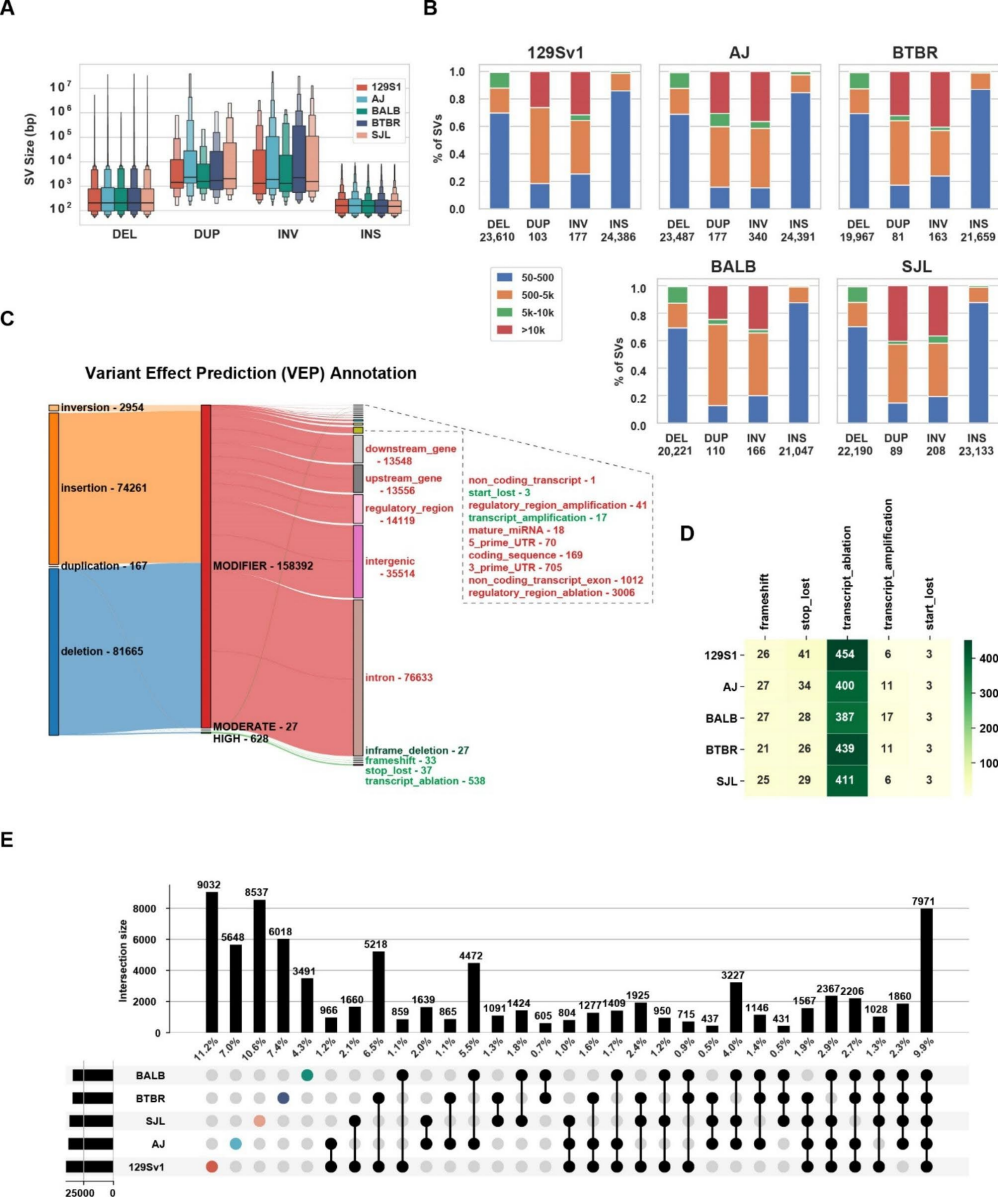
Characterization of SVs within the 129Sv1, A/J, SJL, Balb/c and BTBR genomes. (**A**) The letter-value boxplots [62] show the size distribution of the 4 different types of SVs that are present in the genomes of the 5 strains: DEL, deletions; DUP, duplications; INV, inversions; and INS, insertions. The wide box shows the 25–75% values, while each of the smaller boxes show 12.5% of each data set. (**B**) Each of the four types of SV are categorized according to their size in each strain, and the total numbers of each type of SV is shown at the bottom. **(C)** This Sankey diagram shows the predicted functional consequences for the four different types of SV, which are categorized by their estimated severity (MODIFIER, MODERATE, HIGH). Only 628 SV are predicted to have a high functional impact (green), while most SVs are predicted to have a minor impact. The number of SVs with each type of functional annotation are indicated. (**D**). The number and type of the high impact SVs present in each of the 5 strains are shown. (**E**) This UpSet plot shows unique and shared SVs for each of the 5 strains. In the top graph, each vertical bar represents the number (and percentage) of SVs present in the strain(s) indicated in the intersection matrix, which is located below the top graph. In the intersection matrix, the total number of SVs in each of the 5 strains is indicated by the horizontal bar on the left; each colored dot indicates a single strain; and bars with 2 or more black dots indicate the number of shared SV among the strains indicated by the black dots

*A genome-wide assessment of SV among six inbred mouse strains.* LR genomic sequencing of six inbred mouse strains (BTBR, 129Sv1/J, C57BL/6/J, Balb/c/J, A/J, SJL/J), which was performed using the PacBio Sequel II SMRT Cell system, had an average read length of 15.6 kb and > 40x fold genome coverage (**Table**S1). The LR sequences were aligned to the reference C57BL/6 sequence; and the SVs identified ranged in size from 50 bp to 10 kb. There were 48,292, 48,372, 41,528, 41,415, 5482, 45,148 SVs identified within the 129Sv1, AJ, BALB, BTBR, C57BL/6, and SJL genomes, respectively (Fig. 1A). Since C57BL/6 is the reference sequence, only a very small number of SV were identified in its genome; and relatively few passed subsequent quality control parameters, which is why C57BL/6 SVs were not further analyzed here. For the other five strains, deletions and insertions were the most common type of SV, but duplications and inversions were also present. About 80% of the inversions (median 1551 bp) and 85% of the duplications (median 1695 bp) are over 500 bp in size, while 70% of the deletions (median size 209 bp) and 86% of the insertions (median size 156 bp) are < 500 bp. However, 99% of the deletions and insertions are < 10 kb in size. Although duplications and inversions are rarer than deletions, they are more common among SVs that are > 10 kb in size. (Figs. 1A-B). Most (99%) SVs were within non-coding regions (intergenic, intronic, upstream, downstream, or regulatory), and were predicted to have a minor impact based upon an analysis performed using VEP [28]. However, 628 SVs were predicted to have a major impact by causing the loss of a stop- or start-codon, transcript ablation (most common) or amplification, or a frameshift (Fig. 1 C-D). Since strain-specific SV alleles could be responsible for any of the unique properties exhibited by an inbred strain, we identified 9032, 5648, 8537, 6018, and 3497 SVs that were uniquely present in 129S1, AJ, SJL, BTBR, and BALB/c mice, respectively. Of note, only 9.9% of the SVs are commonly shared by all 5 strains **(**Fig. 1E**)**.

**Fig. 2:**
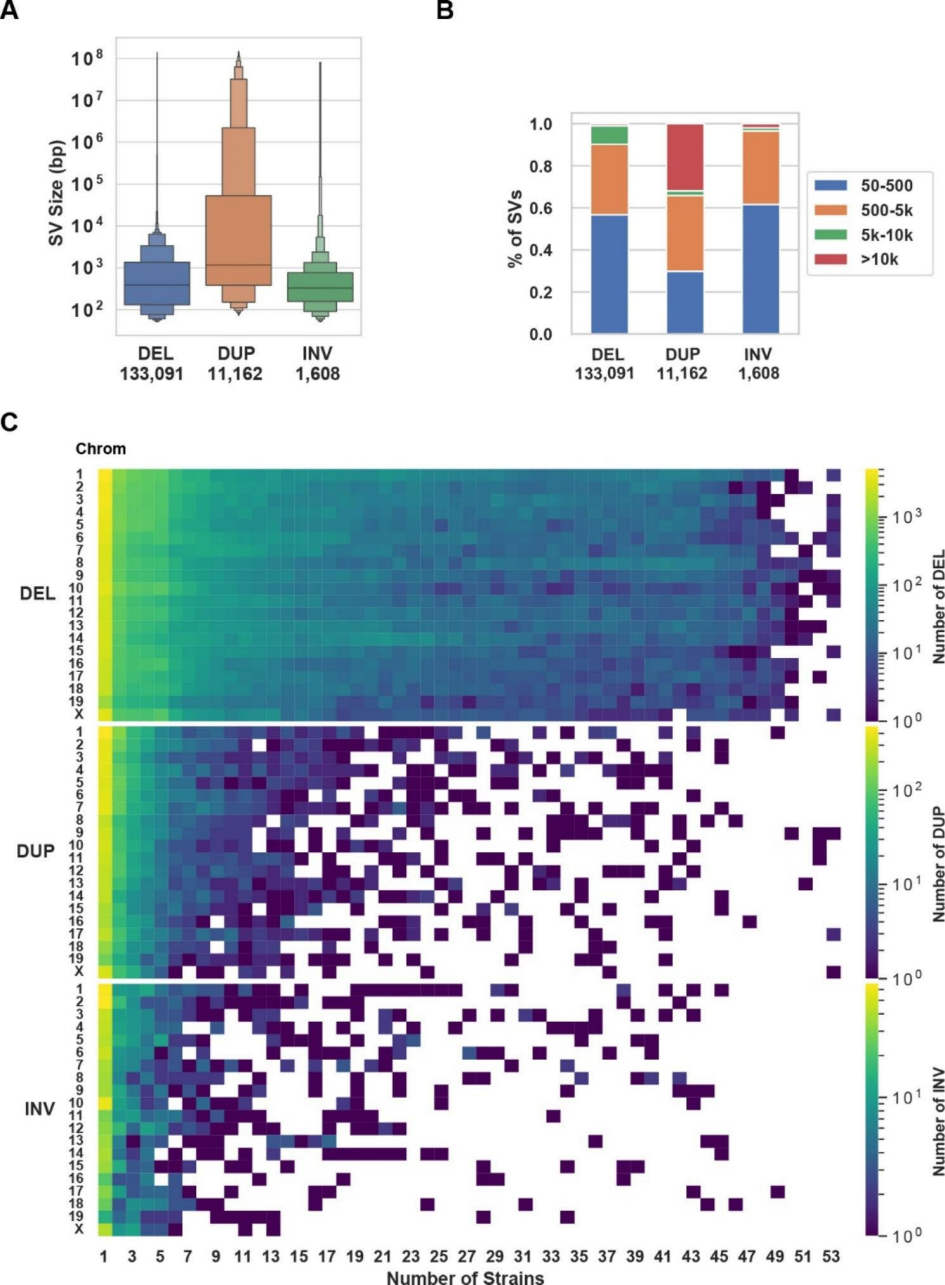
SV within the genome of 53 inbred mouse strains. (**A**) Letter-value boxplots show the size distribution of deletion, duplication and inversion SVs, which have a median length of 337, 680, and 362 bp, respectively. The total number of each type of SV is shown at the bottom. (**B**) The SVs are categorized into four subgroups according to their size: 50–500 bp, 500 bp-5 kb, 5-10 kb, and > 10 kb. Over 90% of the deletions are < 5 kb, 97% of the inversions are < 5 kb, but 19% of the duplications are > 10 kb. (**C**) The number of SVs are categorized according to their type and chromosomal location, and by the number of inbred strains with a strain-shared SV. Each box color indicates the number of each type of SV according to the scale shown at the top. A white area indicates that shared SVs were not found for that number of strains. Deletions are the most common type of SV, and the majority are uniquely present in one strain

**Fig. 3:**
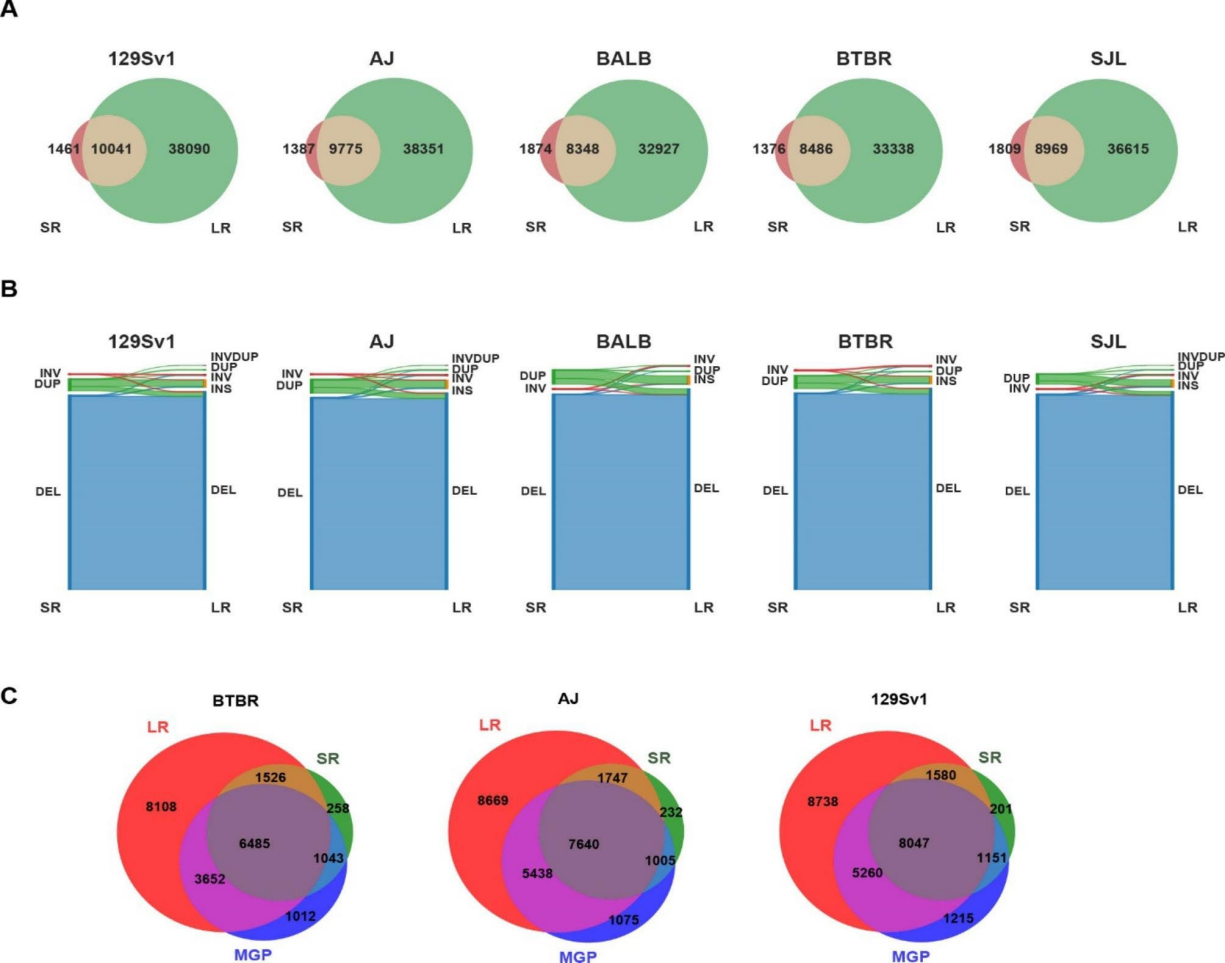
Comparison of SV identified by analysis of SR and LR SV genomic sequence. (**A**) These Venn diagrams show the overlap of the SVs identified by our analysis of LR or SR sequence for the indicated 5 strains. (**B**) These Sankey diagrams indicate the number and type of SR-SVs that were confirmed after analysis of the LR sequence for each strain. Overall, the percentage of SR-SVs that were confirmed by the LR analysis are: 99.4% for DEL, 5% for DUP, and 61.3% for INV. Duplications > 10 kB are the major cause of the discordance between the SR and LR results. (**C**) These Venn diagrams show the overlap of the deletions identified in three inbred strains (BTBR, 129Sv1, and A/J) by our analysis of LR and SR genomic sequence, and with those in the MGP datasets. The number of deletions that were uniquely present in the LR, MGP and SR datasets are indicated in the red, blue, and green areas, respectively. Overall, the LR datasets contain most of deletions found in the SR or MGP datasets, but the LR datasets contain many more deletions than were present in either the SR or MGP datasets

*Comparing SVs identified using LR versus SR sequence.* The SpeedSeq sv pipeline and svtools [29] were used to analyze the available SR genomic sequence for 53 inbred mouse strains (average fold genome coverage 41x, range 19x to 168x) [30]. This analysis identified 133,091 deletions, 11,162 duplications, and 1,608 inversions within their genomes (Fig. 2A-C). Several important observations emerged from analysis of these SVs, which are referred to as **SR-SV**. (i) Although the median size of a duplicated region was 1162 bp, 32% of these were > 10 kb; (ii) deletions were 12-fold more abundant than duplications and are a major contributor to inter-strain differences in SV alleles; (iii) a large percentage of the deletions were strain-specific (Fig. 2C). To assess the quality of the SR-SVs identified using the SR sequence workflow, we assessed their overlap with SVs identified by LR sequence analysis for the 5 strains with available LR sequence. The SR-SVs accounted for only 25% of the SVs identified by LR sequence analysis. Over 85% of SR-SVs overlapped with those identified by LR sequence analysis and the vast majority (99%) of the deletions were similarly classified by the SR and LR sequence analyses (Fig. 3A). However, there were significant differences in the results produced by the two analysis methods. Only 4.7% of the duplications and 60% of the inversions identified by the SR analyses were similarly classified by the LR analysis (Fig. 3B). Since the SR SV pipeline [29] has difficulty identifying insertions, and 32% of the SR duplications are > 10 kb in size (Fig. 3B), it is not surprising that many of the duplications (53–63%) identified by the SR sequence analysis were reclassified as insertions by the LR analysis (**Fig.**S1). We also compared a subset of the SVs (deletions) identified by our LR and SR sequence analyses with those identified by the mouse genome project (MGP) [11] (ftp://ftp-mouse.sanger.ac.uk/). While our SR sequence analysis and the MGP used different analytic methods, there was 81–84% concordance between the SVs identified by the two methods for three inbred strains. However, our LR dataset contained many more (> 8 K) deletions than were present in either the SR or MGP datasets (Supplemental note 1, Fig. 3C). These results indicate that LR sequencing enables many more SVs to be detected and that the SVs were more accurately classified by LR sequencing, which indicates that that LR sequencing is required for a comprehensive characterization of the SVs present in the inbred strain genome.

**Fig. 4:**
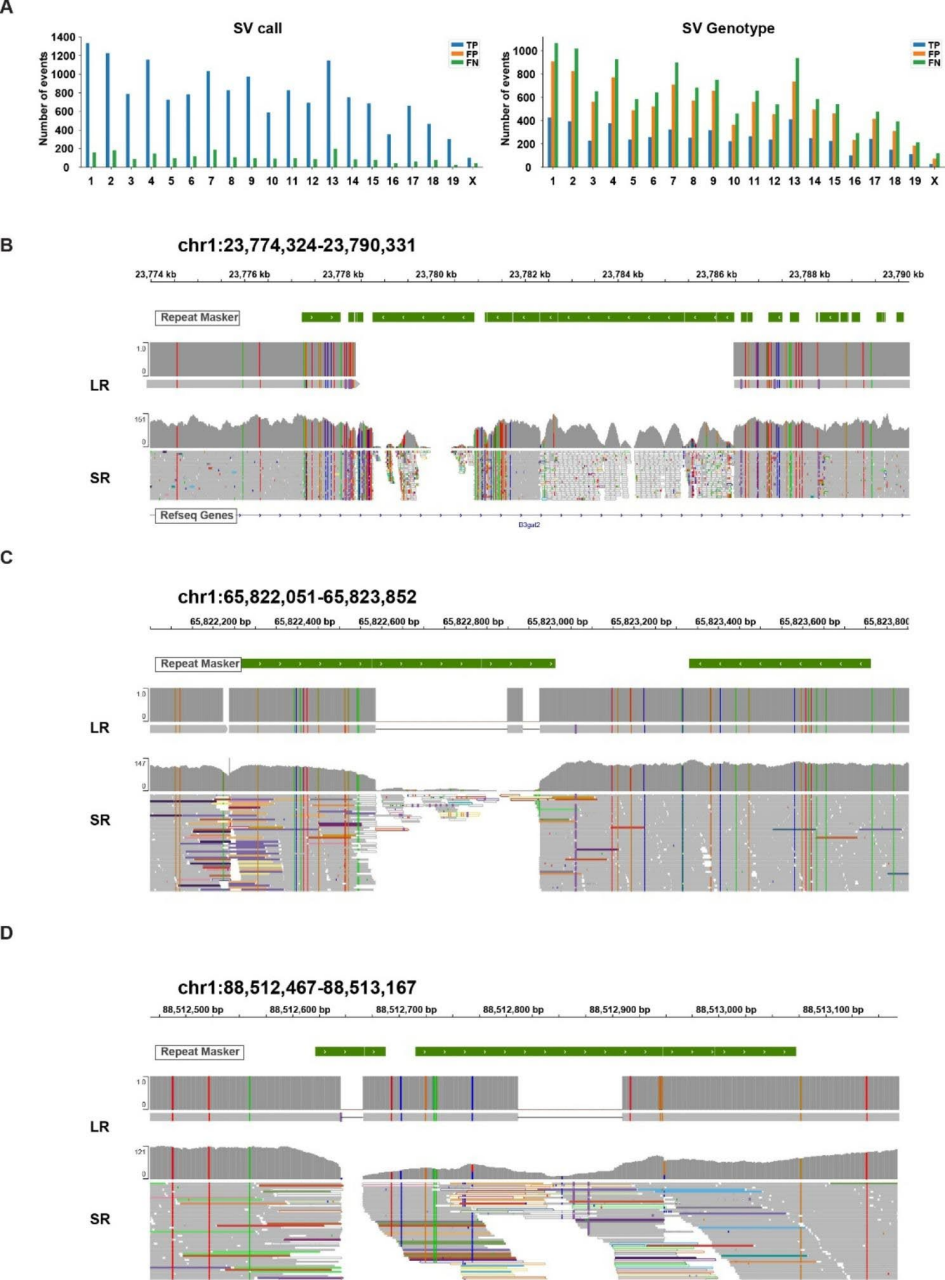
Short-read (SR) sequence analysis has a very limited ability to identify SV present in the genome of inbred strains even when the coordinates for the SV are known. SV were identified by de novo assembly of BTBR LR genomic sequence. These results were compared with the SVs that were identified by analysis of SR BTBR genomic sequence. (**A**) Evaluation of the SVs identified (left panel) and genotyping calls (right panel) by the vg program are displayed by chromosome. For SV calling, 88.3% of known SVs (True Positive, TP) were correctly identified by BTBR SR genomic sequence analysis; there were no False Positive (FP) events; and only 11.6% (False Negatives, FN) of the known SV were missed using the SR sequence. However, only 29% of the known SV were correctly genotyped as homozygous by the SR analysis. (**B-D**) SR alignments for three heterozygous SV were visualized using the integrative genomics viewer. The deletions shown in these 3 examples are homozygous SVs present in BTBR, which were inferred from the *de novo* assembly of the LR BTBR genomic sequence. However, there are SR sequence segments that align with sequences within the deleted region. The repeat masker at the top of each image shows the locations of repeats and low complexity sequence regions, which are the sites that improperly align with some SR segments

**Fig. 5:**
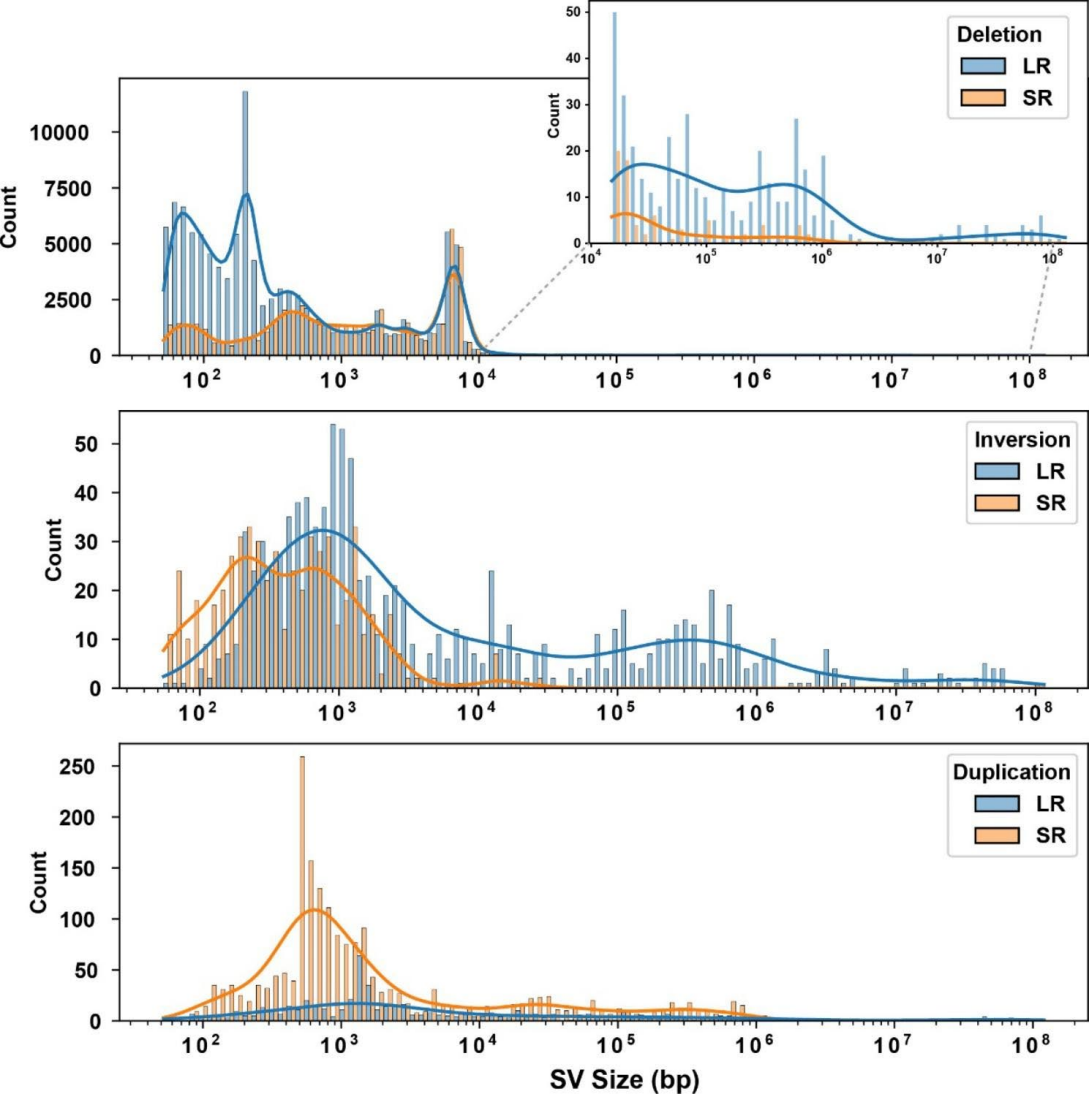
Histograms showing the size distribution of the different types of structural variants (SV) identified by analysis of long read (LR) or short read (SR) genomic sequence data for 5 inbred strains. The lines show the continuous density distribution for each type of SV as determined by Gaussian kernel estimation. Deletions are the most abundant type of SV, and the top graph indicates that many more deletions are identified using LR genomic sequence, especially when the deletion size is either < 1kB or > 10kB. The density lines in the middle graph show that LR sequence analysis also identifies more inversions, especially those with a size > 1kB. In the bottom graph, it appears that more duplications were detected with SR sequence. However, similar to what was observed with deletions (see Fig. 4B-D), the increased number of duplications may result from improper alignment of SR genomic segments, which occurs because of the limitations of SR genomic sequencing technology. Of importance, the small number of very large SVs must be experimentally verified

It was possible that SV identification using SR sequence data could be improved if the genomic coordinates for SVs, which were identified in other trains, was utilized. To test this, 17,503 homozygous SV deletions identified from analysis of the LR BTBR genomic sequence were used as prior information. We then evaluated how many of these deletions would be correctly re-discovered by analysis of SR BTBR genomic sequence, and this analysis was performed using the vg toolkit [31]. We found that 15,444 deletions (88.3%) (i.e., true positives, TP) were correctly identified from the SR sequence analysis; there were no false positive (FP) events; and only 2059 deletions (11.6%) were not rediscovered (i.e., false negatives, FN) from analysis of BTBR SR genomic sequence. However, the ability to distinguish heterozygous from homozygous deletions (i.e., the genotyping performance) was dramatically decreased when SR sequence was analyzed: only 29% of the deletions were correctly genotyped as homozygous (Fig. 4A). Since homozygosity is required for SV identification, the high error rate is caused by deficiencies in genotype calling when SR sequence is analyzed. To investigate the basis for this, the SR alignments for three heterozygous SV were visualized (Fig. 4B-D). The incorrect genotyping calls occurred because some SR sequence segments were falsely aligned (with high mapping quality, MAPQ > 30) within the regions that were actually deleted. A subsequent analysis revealed that these SR segments were improperly aligned to genomic regions that contained repeats or low complexity sequence (Fig. 4B-D**)**. Since the SR (BWA) sequence aligner will search for a ‘best possible match’ for SR segments within genomic regions that contain repeats or low complexity sequence, some SR segments will be falsely aligned to regions that are deleted. Analysis of the size distribution of the different types of structural variants (SV) identified using long read (LR) or short read (SR) genomic sequence data also demonstrate the superiority of using LR genomic sequence data (Fig. 5). LR genomic sequence identified many more deletions (especially when the deletion size is either < 1kB or > 10kB) and many more inversions (especially those with a size > 1kB). It appeared that more duplications were detected with SR sequence. However, analogous to what was observed with the deletion genotyping calls in Fig. 4B-D, the increased number of inversions may result from improper alignment of SR segments when SR sequence technology is used. There are a very small number of SVs, which were not identified by analysis of the LR genomic sequence, that were only identified by SR genomic sequence analysis (Fig. 3A). However, comparison of the corresponding genomic regions for in the SR and LR sequences indicates that the SR-only SVs are likely to be false positives (**Fig.**S2).

*The relationship between SV and SNP alleles.* We also examined the relationship between the 146 K SR-SV identified here and 22 M SNP alleles that we previously identified in the genomes of 53 inbred strains [30]. Just as for a SNP allele, the presence or absence of a SV was treated as an individual allelic variant, even though it impacts > 50 bp. The average distance for a 50% decay in the mean linkage disequilibrium (LD) (${r}^{2}$) that was calculated using both SNP and SV alleles (30–38 kb) was very similar to that when only SNP alleles were used (31–40 kb) (**Fig.**S3). We then examined the relationship between SV and SNP alleles by characterizing LD within localized (± 50 kb) genomic regions; and the strain-unique and shared (i.e., present in ≥2 strains) SR-SVs were separately analyzed. Across all 53 inbred strains, only 3% of strain-unique SVs (1956) are in complete LD with nearby SNP alleles (**Tables**, S2, S3A), while 41% (32,748) of the shared SVs are in complete LD with nearby SNPs. We also analyzed the subset of SVs located within the 21,832 protein-coding genes. Across the 53 inbred strains, a protein coding gene had an average of 4.8 SVs, and each SV is in complete LD (${r}^{2}=1$) with an average of 4.5 nearby SNPs. Thus, irrespective of whether whole genome or intergenic regions were analyzed, SV-SNP allelic relationships are quite similar (**Table**S3B).

Our prior analysis of genome-wide SNP allele relationships separated the 53 inbred strains into four sub-groups [32]. The six strains in sub-group 1 are derived from a C57BL ancestor; sub-groups 2 (17 strains) and 3 (25 strains) contain most of the classical inbred strains; and the five strains in sub-group 4 are wild-derived (**Table**S2). The classical inbred strains in sub-groups 1, 2 and 3 have 2.1-fold more shared SVs than strain-unique SVs, which is probably due to their having shared genomic segments that are derived from ~ four ancestral founders [33, 34] (**Table** S2). In contrast, the wild-derived strains have 2.3-fold more strain-unique than shared SVs, which is consistent with their increased genetic divergence. Overall, the number of SVs uniquely present in the genomes of the five wild-derived strains are 59% of the total number of SVs identified in all 53 strains. As discussed in supplemental note 2, inclusion of the wild-derived (group 4) strains dramatically reduced the LD relationships between SV and SNP alleles among the groups 1, 2 and 3 strains (**Fig.** S4).

**Fig. 6:**
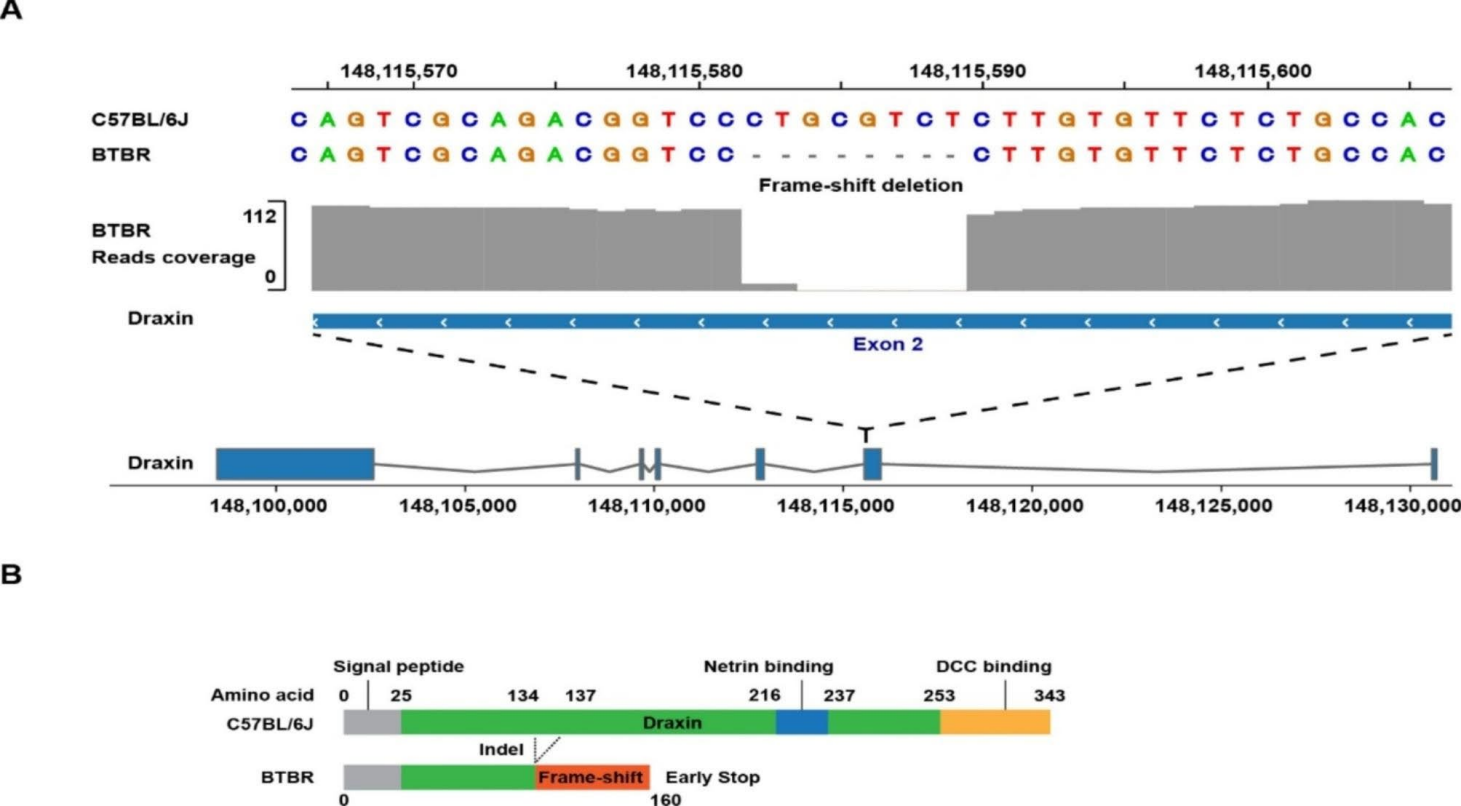
BTBR mice have a non-functional Draxin protein that contributes to the absence of its corpus collosum (CC). **A**) BTBR has an 8 bp deletion at the 3’ end of exon 2 of *Draxin*, which is not present in 52 other strains. **B**) The full length draxin protein has 343 amino acids, but this frameshift deletion generates a termination codon at amino acid 160; this eliminates the Netrin and DCC binding domains from BTBR Draxin that are essential for its neurodevelopmental function. **C**) The CC is partially restored in BTBR mice with a heterozygous knockin (KI) that reverted the 8 bp *Draxin* deletion to wild type (BTBR^*Draxin WT/−*^ KI mice). Coronal (rows 1–2) and horizontal (row 3) images of adult female C57BL/6, BTBR and BTBR^*Draxin WT/−*^ KI mice obtained with a Bruker 11.7-T MRI. Each row represents aligned brain sections obtained from these mice. The CC is within the areas indicated by the red dotted lines. BTBR mice have a complete agenesis of the CC (as indicated by the disconnection between the left and right hemispheres), the CC of C57BL/6 mice is intact, and the CC in BTBR^*Draxin WT/−*^ KI mice was partially restored. **D**) The length of the CC was quantitated along the rostro-caudal axis by analysis of serial aligned coronal sections (n = 3 mice per group). The red dotted lines shown in the top two rows of Fig. 6C outline the CC. The CC length is determined by an automated measurement of the distance between the outer two ends of the CC (excluding gaps) that are shown in the outline. The sites where the BTBR ^*Draxin WT/−*^ KI measurements significantly differ from BTBR (Tukey’s multiple comparison test) are indicated (*, p < 0.05; and **, p < 0.01.The partial correction of the CC in BTBR^*Draxin WT/−*^ KI mice is indicated by the significantly increased length of the CC relative to that in aligned sections from BTBR mice; however, the inter-hemispheric connections in the more rostral and caudal sections of BTBR^*Draxin WT/−*^ KI mice are below those in C57BL/6 mice

*A genetic factor for an ASD-like abnormality of BTBR mice.* Genetic studies have identified multiple BTBR genomic regions that make distinct contributions to their ASD-like abnormalities [23]. Since we now have the BTBR LR genomic sequence, we investigated whether BTBR-specific genetic factors causing its ASD-like abnormalities could be identified. ASD patients have a much higher frequency of highly disruptive mutations (copy number variation [35], premature termination codons (PTC), or frameshift) within neuro-developmentally important genes that are very rare in the general population [36, 37]. Therefore, we sequentially analyzed the BTBR LR sequence, along with the available SR genomic sequence for 52 other inbred strains [30], to identify BTBR-specific SVs and indels that have a high impact on genes expressed in brain. As it turned out, none of the 8 genes with BTBR-specific SVs meeting these criteria had a previously reported connection with ASD (**Table** S4). Since we could not make a compelling case for any of the BTBR-specific SVs, we next examined the BTBR genomic sequence to identify BTBR-unique high impact indels (i.e., SV ≤ 50 bp in size) (Table 1). Of the six genes so identified, an 8-bp frameshift deletion at the end of exon 2 of the *dorsal repulsive axon guidance protein* (*Draxin)* was of particular interest because *Draxin* is located within a previously identified genomic region that contributes to BTBR commissural abnormalities [23]. Moreover, Draxin is a ligand for an axonal guidance receptor DCC [38, 39], and *Draxin* knockout (KO) mice have abnormal development of the CC and forebrain commissures [40]. Developing CC axons are guided to their final destination by midline glial structures [41], which are absent in *Draxin* KO mice [40]. The truncated BTBR Draxin protein is missing key binding domains that are essential for its function in regulating neurite outgrowth (Fig. 6A-B**)**, which makes BTBR mice the equivalent of *Draxin* KO mice [40]. We also identified a BTBR-unique 26 bp frameshift deletion within *Parp10 (ARTD10)* that could possibly also contribute to its ASD-like properties (supplemental note 3, **Fig.** S5), but *Parp10* was not further studied here.

**Table 1:** **BTBR-unique high impact indels.** The gene name, associated transcript; functional consequence, location, BTBR unique allele, and amino acid change(s) (C57BL/6 vs. BTBR) for 6 high impact BTBR-unique indels are shown

Symbol	Consequence	Location	BTBR Allele	C57BL/6	BTBR
*Rnf144a*	splice donor	12:26313980–26,314,017	TCTCTCTCTCTCTCTCTCTCTCTCTCTCTCTCTCTCTCTCTCTCTCTCTCTCTC	-	-
*Parp10*	frameshift	15:76242936–76,242,962	G	AEHRLHGVRL	AX
*Draxin*	frameshift	4:148115582–148,115,591	CT	KRRR	KX
*Vmn2r29*	frameshift	7:7247280	TT	M	NX
*Vmn2r50*	frameshift	7:10048203–10,048,204	C	G	X
*Zfp108*	splice acceptor	7:24255827–24,255,830	CCCCC	-	-

To determine whether the *Draxin* indel contributed to its ASD-like neuroanatomic abnormalities, a knockin (KI) mouse with a heterozygous reversion of the 8 bp *Draxin* deletion to wild type was generated on an otherwise BTBR genetic background (BTBR^*Draxin WT/−*^ KI) by CRISPR engineering (**Fig.** S6). A quantitative analysis of the length of the CC was performed by analyzing MRI images obtained from BTBR, C57BL/6 and BTBR^*Draxin WT/−*^ KI mice, which were obtained from multiple sectional images along the rostral caudal axis. The CC thickness in all sections obtained from BTBR mice were all below those of C57BL/6 mice. However, CC thicknesses in multiple sections obtained from BTBR^*Draxin* WT/−^ KI mice were like those of C57BL/6 mice, which indicates that the CC was partially restored in BTBR^*Draxin* WT/−^ KI mice (Fig. 6C-D). However, the inter-hemispheric connections in the more rostral and caudal sections of BTBR^*Draxin WT/−*^ KI mice are below those in C57BL/6 mice.

## Discussion

Several important features about the impact that SVs have on the pattern of genetic variation in the genome of inbred strains are revealed by our analysis. (i) SV are abundant (average 4.8 per gene), which indicates that they are highly likely to impact genetic traits. (ii) SV and SNP alleles are more concordant among the classical inbred strains than among wild-derived strains, but we have a very limited ability to infer whether a known SV is present by analysis of nearby SNP alleles. Moreover, it is difficult to detect strain-specific SVs by analysis of SR sequence (discussed in supplemental note 4). (iii) LR sequencing of additional strains is needed to produce a more complete map of the pattern of genetic variation among inbred mouse strains. The results that we obtained from analysis of murine genomic sequence are consistent with a recent comparison of the frequency of human SVs that could be discovered using LR and SR sequence, which also found that only 29% of human LR-SVs could be identified by analysis of SR sequence [42].

Prior analyses of BTBR mice have identified many genes and potential pathways that could contribute its ASD-like properties [22–25, 27, 43, 44], and alterations in intestinal bacteria have been implicated as a contributor to their social behavior deficits [45]. However, because the prior studies did not have access to the complete BTBR genomic sequence, they could not generate specific hypotheses about the genetic basis for its ASD-like features. By sequentially analyzing the SVs and indels present in the BTBR genome, we identified a BTBR-unique indel that contributes to its ASD-like features. Several features of the BTBR *Draxin* indel are consistent with the results obtained from GWAS performed on several human ASD cohorts and from BTBR intercross progeny. (i) ASD patients have a much higher frequency of disruptive mutations in neurodevelopmentally important genes that are very rare in the general population [36, 37]. (ii) Human GWAS have associated *DRAXIN* alleles with susceptibility to schizophrenia (rs4846033, p-value 4 × 10^− 6^, intergenic variant) [46] and ASD (rs12045323, 7 × 10^− 6^, intronic variant) [47]. (iii) Murine *Draxin* is within a chromosomal region that was previously identified as containing a major contributor to its commissural abnormalities [23]. Analysis of the BTBR ^*Draxin WT/−*^ KI mouse confirmed that the *Draxin* deletion contributes to its commissural abnormalities. Since reversion of the BTBR *Draxin* deletion only partially corrected the commissural abnormalities, there must be other BTBR-unique genetic factors that contribute to its ASD-like properties. This result is consistent with the fact that the CC defects in *Draxin* KO mice were quite variable [39, 40], while the CC is completely absent in BTBR mice. Also, analysis of BTBR intercross progeny have indicated that multiple chromosomal regions contribute to its abnormal commissural morphology, and these are distinct from the genomic regions that contribute to its altered behavior [23]. The fact that a human ASD-related phenotype in mice could have an oligogenic basis is not unexpected. For example, cardiac abnormalities in a murine model of a human congenital cardiomyopathy did not appear until three different causative alleles – each homologous to a human disease-causing allele - were CRISPR engineered into three different murine genes [48]. Of importance, CRISPR methodology enables us to genetically engineer changes into the genome of any inbred strain, and tissue can be readily obtained for analysis at various developmental stages. Therefore, subsequent analyses of the effect of the *Draxin* indel along with other BTBR-unique genetic factors have on mouse neurodevelopment and behavior should provide new insight into the pathogenesis of ASD. More broadly, these results demonstrate how obtaining a more complete picture of the pattern of genetic variation among inbred mouse strains can facilitate genetic discovery.

### Conclusion

A more complete map of the pattern of genetic variation among inbred strains, which is produced by LR genomic sequencing of the genomes of additional inbred strains, could facilitate genetic discovery when murine models of human diseases are analyzed.

**Note added in proof**. While this work was ongoing, a paper by Morcom et al. [49] analyzed (C57BL/6 x BTBR) F2 intercross progeny and identified the 8-bp deletion in *Draxin* as the probable cause for the absence of the CC.

## Materials and methods

*Animal experiments*. All animal experiments were performed according to protocols that were approved by the Stanford Institutional Animal Care and Use Committee. All mice were obtained from Jackson Labs, and were used at 10–12 weeks of age, and the results are reported according to the ARRIVE guidelines [50].

*LR DNA sequencing.* DNA was extracted from tails obtained from male 129Sv1/J, BTBR/J, SJL/J, A/J, Balb/c/J, and C57BL/6J mice (n = 1 per strain) using the Qiagen QIAmp DNA Kit (Qiagen, Hilden, Germany). The DNA concentration was measured using the Qubit 2.0 Fluorometer (Life Technologies, Carlsbad, CA, USA); and DNA purity and integrity were checked using a Nanodrop and by pulsed field gel analysis, respectively; and 50 pM DNA concentrations were used for LR sequencing of each strain. Biosciences (PacBio, Menlo Park, CA) LR SMRTbell libraries (~ 20 kb) were prepared using Blue Pippin size selection according to the manufacturer’s protocol (Sequel II Sequencing Kit 2.0 with Sequel II DNA Polymerase 2.0), and LR sequencing was performed using the PacBio Sequel II SMRT Cell system.

*Data processing and SV identification.* The PacBio raw bam format files were converted to the fastq format using the bam2fastx method using the default commands. CoNvex Gap-cost alignMents for LRs (ngmlr v0.2.7) [51] were used to align the raw data to the reference genome (mm10) using the aligner commends (-x pacbio -i < default> -R < default> -t 15). The alignment output files were sorted and converted into bam format files with the samtools *view* commend [52]. SV identification for each sample was performed using Sniffles (v.1.0.12b) with parameters: -s 8 -l 50 --min_homo_af 0.7 --min_het_af 0.25 --genotype --cluster [51]. To perform the downstream functional analysis of SVs present in each strain, Sniffles SV results were filtered to retain only those genomic positions with > 50 bp changes, which were homozygous alternate calls and contained PASS tag. To identify shared and strain-specific SVs, a merged dataset was also assessed using Sniffles’s population calling pipeline (parameters: -s 8 -l 50 --min_homo_af 0.7 --genotype --cluster --Ivcf ). SVs of the merged callset were filtered if a certain SV was a translocation, or if there was a homozygous alternate call in the control strain C57BL6 (reference genome), or no homozygous alternate call across the 6 new strains.

*Manual inspection*. The output file from the LR SV caller manual inspected using a previously described method [53] to ensure that all SV calls are true. In brief, Samplot (https://github.com/ryanlayer/samplot) was used to virtually inspect all identified SVs for each LR genomic sequence analyzed. This method analyzes variant (vcf) and alignment (bam) files, and then outputs a graphical representation for each SV, where the y-axis shows the read depth and x-axis shows the start and end points of each SV. The SV calls are displayed as the difference in the read depth between the genomic coordinates for a SV and the surrounding genomic region. On the rare occasion when a graph revealed that the sequencing depth within a region identified as a SV was not different from its surrounding genomic region, the SV call was rejected.

*Genomic feature annotation.* To assess the impact of a SV on a gene, the filtered and merged callset for the 6 strains was annotated using the Ensemble Variant Effect Predictor (VEP) program [28]. Based upon the intersection between a SV and a type of genomic region, VEP annotates the effect of the SV, and we focused upon the SV that impacted coding exons. Annotations for genomic repeat elements were obtained using the *RepeatMasker* software package [54]. To identify genes that were expressed in brain, expression data was retrieved from the Expression Atlas database [55]. Genes whose expression level was ≥ 10TRM, which is the basal level used by this database, were identified as genes that were expressed in brain.

*SR-SV analysis.* The SR SV dataset was constructed using the genome sequence of 53 inbred mouse strains [30]. Data were realigned to GRCm38 using the SpeedSeq (v0.1.2) realign pipeline, and then SV analysis was performed using by SpeedSeq sv pipleline (Lumpy v0.2.13, CNVnator v0.4.1, SVTyper v0.7.0) with extra parameters: -d -P -g -k. The individual SV data for the 53 strains were merged, re-genotyped, copy-number annotated, and pruned using the svtools (v0.4.0) workflow. To obtain high quality SR-SV calls, we searched for SVs with the following parameters: SV size > 50 bp, homozygous rate > 94% (i.e., ≥ 50 strain genotypes are homozygous); at least 1 strain’s genotype is homozyous alternate; deletions < 1000 bp required the support of at least 1 split read; inversion’s MSQ (mean sample quality) > 150; QUAL > = 20.

*Comparing SV identified by analysis of SR and LR BTBR genomic sequence.* The variant graph toolkit [31, 56] was used to compare the deletions identified by analysis of SR sequence with those identified using LR sequence. In brief, we used 17,503 homozygous deletions (size < 10 kb, allele frequency > 0.8), which were identified from analysis of BTBR LR sequence, to construct a variant graph. Then, the BTBR SR sequence were aligned to the variant graph. We then performed SV calling and genotyping using the default parameters specified in vg toolkit; and the default criteria specified in the sveval package [56] was used to evaluate whether a deletion could be correctly predicted from SR sequence analysis by disregarding the genotype call. The 17,503 deletions identified from analysis of LR BTBR genomic sequence were used as the prior information, which served as the gold standard for evaluating a SR deletion prediction. Then, a true positive (TP) SR deletion was defined as one that covered > 50% of the LR deletion region and had > 10% overlap of SR deletion. A false negative (FN) deletion was one where the predicted SR deletion region had < 50% overlap with the LR deletion, and a false positive (FP) deletion had < 10% reciprocal overlap. For evaluating the genotype predictions, we separately evaluated the genotype calls, which indicated whether the deletions were identified as heterozygous or homozygous, using the same criteria. For each genotype, the overlaps are used to build a bipartite graph, each variant call is matched with a LR variant using bipartite clustering. All variant matches are considered as TP, and the remainder are errors (FP) or false negatives (FN). For some comparisons, the deletions contained within the mouse genome project (MGP) dataset [11] (ftp://ftp-mouse.sanger.ac.uk/) were downloaded, and the MGP deletions were compared with those in our LR and SR datasets. A genomic interval- based comparison of the deletions present in these datasets was performed using Bedtools software using the default parameters [57].

*Evaluation of LD between SV and SNP alleles.* The relationship between SVs (n = 146 K) identified from analysis of SR genomic sequence and previously identified SNPs (22 M) in the genomes of 53 inbred mouse strains [30] was investigated. For these analyses, a SV was treated as an individual allelic variant (even though it impacts > 50 bp), which was just the same as a SNP allele. The rate of decay in the linkage disequilibrium (LD) between identified alleles was analyzed using PopLDdecay (v3.41) [58]. Pairwise LD statistics (${r}^{2}$) for SV and nearby SNPs (within ± 50 kb) were calculated using PLINK 1.90 [59] for all 53 inbred mouse strains. Because LD is the nonrandom correlation of relationship between alleles present at different loci, which is affected by non-random mating that does not occur among the inbred strains, LD relationships will mostly reflect the strain genealogy. We previously found that the 53 mouse strains with available genomic sequence could be separated into four sub-groups based upon their genome-wide genetic relatedness [60]. To better characterize the relationship between SVs and nearby SNP alleles, we also investigated whether SV alleles are in complete LD (${r}^{2}$=1) with nearby SNP alleles (within 50 kb) within the strains in the 4 subgroups. The significance of the LD between SV and SNP alleles is obtained by calculating $N{r}^{2}$, which follows a Chi-square distribution with one degree of freedom ($N{r}^{2}\tilde{\chi }_{1}^{2}$), where *N* is the number of strains. Thus, the p-values required for a SV to be perfectly linked with a SNP in the whole strain panel and or among each of the 4 sub-groups of the 53 inbred strains are ${p}_{53}=3.3E-13$, ${p}_{6}=0.014$, ${p}_{17}=3.7E-5$, ${p}_{25}=5.7E-7$ and ${p}_{5}=0.025$, respectively.

*Generation of BTBR KI mice with wild type Draxin*. Three-week old BTBR female mice were super-ovulated by intraperitoneal injection of pregnant mare’s serum gonadotropin and human chorionic gonadotropin. These mice were then paired with BTBR males to generate fertilized embryos, and pronucleus (PN) stage embryos were collected [61]. Cas9, an sgRNA (GAAACGTGGCAGAGAACACA) and a ssODN (GGTCTCCCGCTTGGGAGAGG GTGAAGAAACGTGGCAGAGAACACAAGAGACGCAGGGACCGTCTGCGACTGCACCGAGGTAGCTGGAGACCTGGGGGGAGGAGGGAACTACA) were then electroporated into PN embryos using a NEPA21 electroporation system. The ssODN was designed to revert an 8 bp deletion in *Draxin*, which is present in BTBR mice, to wildtype. Healthy embryos were transferred into the oviducts of pseudo-pregnant recipient females. Genomic DNA from the pups were screened by PCR amplification followed by BsmBI-v2 digestion and sequencing (Fig. S5). The amplification primers used are: Drax-F (CACTCATGATGCTGGTTTTCTTTCAG) and Drax-R (CTAAGGGAGCAGAACTTCTATGTCAG). Sequencing of the PCR product from a BTBR *Draxin* KI founder (pup #3) confirmed that the 8 bp deleted sequence was reverted to wildtype. Pup#3 was backcrossed with BTBR to obtain a heterozygous BTBR *Draxin* KI on a clean BTBR background before it was experimentally analyzed. When the experimental analyses were completed, euthanasia was carried out by the method of CO_2_ asphyxiation.

*MRI analyses*. The brains of age-matched adult female C57BL/6, BTBR and BTBR^*Draxin WT/−*^ KI mice (n = 3/group) were examined by in vivo MRI using a high-field 11.7T MRI scanner (Bruker Corp, Billerica, MA), which is housed at the Stanford Center for Innovation in In vivo Imaging (SCi^3^) facility. Hence, 9 mice were used in these studies. All mice were anesthetized with 1.5–1.75% isoflurane that was administered by nose cone throughout the session. Their body temperatures were supported with a 40^o^ C warm water recirculation system, while their respiratory rates were continuously monitored. Anatomical images were acquired using T2-weighted turbo-RARE acquisition (T2 TurboRARE) with the following parameters: repetition time (TR) = 4000 ms, echo time (TE) = 50 ms, flip angle = 90 degrees, field of view 20 × 20 mm, image size 256 × 256, slice thickness = 0.5 mm. One set of slices were obtained in the axial view with the first slice starting at the rostral-most extension of the prefrontal/motor cortex, while the olfactory bulb was excluded. Another set of slices were obtained in coronal view with the first slice starting at the surface of the skull. The DICOM files obtained were processed using Osirix software (Pixmeo SARL, Bernex, Switzerland). The structures of the corpus callosum were manually labelled by an experimenter who was blinded to the genotype of mice. The measurements between the outer ends of the corpus collosum (excluding gaps) were made using the Osirix software on a continuous series of 9 slices (located from + 0.62 mm to -3.38 mm relative to the Bregma), which were aligned across the different groups of mice. The data were analyzed using Prism 9.1.0 (GraphPad Software, Inc. La Jolla, CA) with a 2-way repeated ANOVA measures using Tukey’s multiple comparison test; and the section-series plots were graphed as in Fig. 5D.

## Electronic supplementary material

Below is the link to the electronic supplementary material.

**Additional file 1:Table S1.** The SVs observed at each level of analysis. **Table S2.** The 53 inbred strains with available genomic sequence were divided into the four sub-groups based on their pattern of genome-wide allelic sharing that are shown below [2]. **Table S3.** The numbers of SVs identified using short range sequence in 53 inbred strains. **Table S4.** BTBR-unique SVs. **Figure S1.** Comparison of the type of SV identified by SR and LR sequence analysis. **Figure S2.** Three examples of false positive SR-only homozygous SV calls in the 129S1, BTBR and A/J genomes are shown in panels A-C, respectively. **Figure S3.** A graph of linkage disequilibrium (LD) decay for 53 inbred mouse strains using alleles that were identified using SNPs alone (black), or those after both SV and SNP alleles were analyzed (red). **Figure S4. **LD plots characterizing the relationship between SV and SNP alleles within the 40.5 KB Fam20b genomic region. **Figure S5. **BTBR mice produce a non-functional Parp10 protein. **Figure S6.** CRISPR-engineering produces a heterozygous BTBR KI mouse (BTBR Draxin WT/- KI) with a reversion of the 8 bp deletion in exon 2 of Draxin to wild type.

## Data Availability

The data supporting this manuscript, which are the LR sequence data files, are available at the NCBI SRA database. http://www.ncbi.nlm.nih.gov/bioproject/788143.; and the SNP and SV data is available at the Mouse Phenome Database (GenomeMUSter https://mpd.jax.org/genotypes).

## Abbreviations

ASD: Autism Spectral Disorder
CC: corpus collosum
DS: Dravet’s syndrome
ECM: extracellular matrix
KI: knockin mouse
KO: knockout mouse
LOF: loss of function
LR: long read
MGP: Mouse Genome Project
MRI: magnetic resonance imaging
SNP: single nucleotide polymorphism
SR: short read
SR-SV: structural variants identified using SR sequence
SV: structural variant
