# Supplementary material for: Analysis of structural variation among inbred mouse strains
Source: BMC Genomics. 2023 Mar 2;24:97. doi: 10.1186/s12864-023-09197-5 (PMC9983223; doi:10.1186/s12864-023-09197-5)
Supplement: Supplementary file 1 — Additional file 1:Table S1. The SVs observed at each level of analysis. Table S2. The 53 inbred strains with available genomic sequence were divided into the four sub-groups based on their pattern of genome-wide allelic sharing that are shown below [2]. Table S3. The numbers of SVs identified using short range sequence in 53 inbred strains. Table S4. BTBR-unique SVs. Figure S1. Comparison of the type of SV identified by SR and LR sequence analysis. Figure S2. Three examples of false positive SR-only homozygous SV calls in the 129S1, BTBR and A/J genomes are shown in panels A-C, respectively. Figure S3. A graph of linkage disequilibrium (LD) decay for 53 inbred mouse strains using alleles that were identified using SNPs alone (black), or those after both SV and SNP alleles were analyzed (red). Figure S4. LD plots characterizing the relationship between SV and SNP alleles within the 40.5 KB Fam20b genomic region. Figure S5. BTBR mice produce a non-functional Parp10 protein. Figure S6. CRISPR-engineering produces a heterozygous BTBR KI mouse (BTBR Draxin WT/- KI) with a reversion of the 8 bp deletion in exon 2 of Draxin to wild type. [file 12864_2023_9197_MOESM1_ESM.docx]

**Supplemental Information**

Analysis of Structural Variation Among Inbred Mouse Strains

*Supplemental note 1: Comparison of SVs identified by different methods.* To examine the level of concordance between the SVs present in different datasets, we also compared the SVs identified by our LR and SR sequence analyses with those identified by the mouse genome project (MGP) [1] (<ftp://ftp-mouse.sanger.ac.uk/>). For these comparisons, we examined the overlap for SVs present in all three datasets, which were the deletions identified in BTBR, AJ and 129Sv1 mice (**Fig. 3C**). The MGP dataset, which evaluated SR sequence but used multiple methods for identifying SV, contained a slightly larger number of deletions (BTBR, 12,167; AJ, 15,082; 129Sv1, 15,559) than were present in our SR dataset. Nevertheless, there was a high level of concordance between the deletions present in the SR and MGP datasets: 81% for BTBR, 81% for AJ, and 84% for 129Sv1. However, our LR dataset contained most of the deletions (BTBR,54%; AJ, 63%; 129S1, 63%) that were present in both the SR and MGP datasets (Fig. 3C), and many more deletions (BTBR, 8101; AJ, 8669; 129S1, 8738) than were present in either the SR or MGP datasets. These comparisons indicate that that LR sequencing is required for a comprehensive characterization of the SVs present in the inbred strain genome.

*Supplemental note 2: The effect of strain subgroups.* Analysis of genome-wide SNP allele relationships separated the 53 inbred strains into four sub-groups [2]. The six sub-group 1 strains are derived from a C57BL ancestor; sub-groups 2 (17 strains) and 3 (25 strains) contain most of the classical inbred strains; and the five sub-group 4 strains are wild-derived (**Table S2**). We identified 11443, 46323, 40244 and 114086 SR-SVs within the genomes of the 53 inbred strains in sub-groups 1-4, respectively (**Table S3A**). We examined the LD between the SR-SV and SNPs within strain sub-groups, and the LD relationships were quite dependent upon whether all 53 strains or if subgroups of the strains are analyzed. Over 75% or 84% of the strain-unique SVs are in perfect LD with ≥1 nearby SNP when the sub-group 1-3 stains and subgroup 4 strains are separately evaluated, respectively. Inclusion of the group 4 strain-unique SVs dramatically reduced the LD relationships among the group 1-3 strains. Within each of 4 sub-groups, >71% of shared SVs are in complete LD with nearby SNPs; while across all 53 inbred strains, about 41% (32748) are in complete LD with nearby SNPs. Among the subset of SVs within the 21,832 protein-coding genes, sub-group 1 strains have the lowest number (mean 2.1) of SVs per gene; sub-group 2 and 3 strains have 2.5 SVs per gene on average, and 2 of them are completely linked ($r^{2}=1$) with adjacent SNPs; and sub-group 4 strains have more SVs per gene (mean 4.1). **Fig. S3** illustrates how the relationship between SV and SNP alleles within a gene is affected by whether all strains or subgroups of the strains are analyzed.

*Supplemental note 3: A Parp10 frameshift deletion*. We also identified BTBR-unique 26 bp frameshift deletion in exon 2 of *Parp10 (ARTD10),* which generates a termination codon after amino acid 66. The truncated BTBR protein lacks the catalytic and other domains of Parp10 that are essential for its function (**Fig. S4).** *Parp10,* which is located within an interval on chromosome 15 that contributes to BTBR commissural abnormalities [3], is a mono-ADP ribosylation (marylation) enzyme. Because of its many substrates, it is involved in transcriptional regulation, DNA damage repair, and other cellular processes [4, 5]. While a functional role for Parp10 in ASD has not been directly demonstrated, some evidence suggests that it could play a role. Through marylation of protein kinase C (PKC), Parp10 alters the activity of a voltage-gated potassium channel (Kcna1, Kv1.1), which regulates hippocampal neuron excitability [6]; and Kv1.1 has been shown to modulate ASD-related behaviors in mice with another ion channel mutation [7]. Also, a patient with a homozygous *PARP10* deficiency had neuro-developmental delay and defective DNA repair [8]. PARP10 has been shown to regulate NF-κB [9] and Wnt signaling [10], which are pathways that are known to affect neurodevelopment.

*Supplemental note 4: Can SV be imputed from analysis of SR sequence?* Given that we now have LR sequence data available for only 5 strains, a major question is whether SVs can be characterized for the other inbred strains using SR sequence data? Our results indicate that it is possible to predict whether a known SV (i.e., one identified from analysis of LR sequence data) is present in other strains based upon analysis of nearby SNP alleles. However, since only 24% of SVs are in complete LD with nearby SNPs across the 53-strain panel, the level of certainty for SNP allele-based SV prediction is quite limited. Even when the analysis is confined to SV predictions within a strain subgroup, only 41% of shared SVs are in complete LD with nearby SNPs. Thus, the ability to impute whether SVs are present based upon nearby SNP alleles is limited, and this holds true even when the analysis is confined to the classical inbred strains. Our results also indicate that it can be difficult to identify sites with strain-unique SVs using only SR sequence data. Although >75% of the strain-unique SVs are linked with ≥ 1 adjacent SNP within each of the 4 strain subgroups, only 3% of strain-unique SVs have perfect LD with nearby SNP alleles in the 53-strain panel. The improvement of within group predictions results from the fact that each subgroup contains either closely related strains (subgroups 2 and 3) or has limited variation due to it having a small number of strains (subgroups 1 and 4). Nevertheless, the significant uncertainty associated with imputed SV limits their utility.

**Supplemental References**

1. Doran AG, Wong K, Flint J, Adams DJ, Hunter KW, Keane TM: **Deep genome sequencing and variation analysis of 13 inbred mouse strains defines candidate phenotypic alleles, private variation and homozygous truncating mutations.** *Genome Biol* 2016, **17:**167.

2. Wang M, Fang Z, Yoo B, Bejarano G, Peltz G: **The Effect of Population Structure on Murine Genome-Wide Association Studies.** *Frontiers in Genetics* 2021, **In press**.

3. Jones-Davis DM, Yang M, Rider E, Osbun NC, da Gente GJ, Li J, Katz AM, Weber MD, Sen S, Crawley J, Sherr EH: **Quantitative trait loci for interhemispheric commissure development and social behaviors in the BTBR T(+) tf/J mouse model of autism.** *PLoS One* 2013, **8:**e61829.

4. Kaufmann M, Feijs KL, Luscher B: **Function and regulation of the mono-ADP-ribosyltransferase ARTD10.** *Curr Top Microbiol Immunol* 2015, **384:**167-188.

5. Feijs KL, Verheugd P, Luscher B: **Expanding functions of intracellular resident mono-ADP-ribosylation in cell physiology.** *FEBS J* 2013, **280:**3519-3529.

6. Tian Y, Korn P, Tripathi P, Komnig D, Wiemuth D, Nikouee A, Classen A, Bolm C, Falkenburger BH, Luscher B, Grunder S: **The mono-ADP-ribosyltransferase ARTD10 regulates the voltage-gated K(+) channel Kv1.1 through protein kinase C delta.** *BMC Biol* 2020, **18:**143.

7. Indumathy J, Pruitt A, Gautier NM, Crane K, Glasscock E: **Kv1.1 deficiency alters repetitive and social behaviors in mice and rescues autistic-like behaviors due to Scn2a haploinsufficiency.** *Brain Behav* 2021**:**e02041.

8. Shahrour MA, Nicolae CM, Edvardson S, Ashhab M, Galvan AM, Constantin D, Abu-Libdeh B, Moldovan GL, Elpeleg O: **PARP10 deficiency manifests by severe developmental delay and DNA repair defect.** *Neurogenetics* 2016, **17:**227-232.

9. Verheugd P, Forst AH, Milke L, Herzog N, Feijs KL, Kremmer E, Kleine H, Luscher B: **Regulation of NF-kappaB signalling by the mono-ADP-ribosyltransferase ARTD10.** *Nat Commun* 2013, **4:**1683.

10. Feijs KL, Kleine H, Braczynski A, Forst AH, Herzog N, Verheugd P, Linzen U, Kremmer E, Luscher B: **ARTD10 substrate identification on protein microarrays: regulation of GSK3beta by mono-ADP-ribosylation.** *Cell Commun Signal* 2013, **11:**5.

**Table S1. The SVs observed at each level of analysis.** SVs were identified from LR sequence data obtained from 6 inbred strains. LR sequencing produced on average mean read length of 15.65 kb and an average sequence depth of 40x. The numbers of SV identified and those remaining after quality control steps is shown for each strain. The C57BL/6 (reference) genome has very few SVs because it is the reference genome used for this analysis; and consequently, it does not have any strain-specific SV alleles. In contrast, the 129Sv1 genome has the greatest number of strain-specific SVs.

|  | **129Sv1** | **AJ** | **BALB** | **BTBR** | **C57BL/6** | **SJL** |
| --- | --- | --- | --- | --- | --- | --- |
| Mean read length (bp) | 16939 | 15770 | 14162 | 15872 | 16716 | 14453 |
| Breath of coverage (%) | 93.57 | 96.55 | 96.61 | 95.97 | 94.04 | 96.56 |
| Depth of coverage (X) | 42.09 | 42.69 | 37.50 | 38.63 | 40.01 | 39.19 |
| # SV (total) | 68977 | 66903 | 59703 | 59933 | 8850 | 66787 |
| # SV (filtered) | 48292 | 48372 | 41415 | 41528 | 5482 | 45148 |
| Strain Specific | 9032 | 5648 | 3491 | 6018 | 0 | 8537 |

**Table S2.** The 53 inbred strains with available genomic sequence were divided into the four sub-groups based on their pattern of genome-wide allelic sharing that are shown below [2].

| Group | Number of Strains | Strain List |
| --- | --- | --- |
| 1 | 6 | B10, C57BL6NJ, C57BL10J, C57BRcd, C57LJ, C58 |
| 2 | 17 | 129P2, 129Sv1, 129S5, BPL, BPN, BTBR, CEJ, ILNJ, KK, LPJ, NZB, NZO, NZW, PJ, RBF, SMJ, WSB |
| 3 | 25 | A/J, AKR, BALB, BUB, C3H, CBA, DBA, DBA1J, FVB, LGJ, MAMy, MRL, NOD, NON, NOR, NUJ, PLJ, RFJ, RHJ, RIIIS, SEA, SJL, ST, SWR, TALLYHO |
| 4 | 5 | CAST, MOLF, PWD, PWK, SPRET |

**Table S3.** (**A**) The numbers of SVs that were identified using SR sequence in 53 inbred strains and those within each of four sub-groups of strains are shown. SVs are categorized as either strain-unique (present in only one strain) or strain-shared (present in >2 strains). The number of SVs that are in perfect linkage disequilibrium (LD) ($r^{2}=1$) with nearby SNPs (within ± 50 kb) are shown for each strain group. The mean and median numbers of nearby SNPs that are in complete LD ($r^{2}=1$) with a SV are also shown. (**B**) The same analysis was also performed for SV within the 21,832 protein coding genes in the mouse genome. For each gene within the subset of genes that contain a SV, the average number of SVs that are in perfect linkage disequilibrium (LD) ($r^{2}=1$) with nearby SNPs (within ± 50 kb) and the average number of SNPs that are completely linked with a SV are shown for each group of strains.

**A**

|  | SV Type | # SVs | # SVs in LD with nearby SNPs | Median # SNPs in LD with nearby SV | Mean # SNPs in LD with nearby SV |
| --- | --- | --- | --- | --- | --- |
| 53 strains | unique | 65992 | 1956 | 2 | 4.0 |
|  | shared | 79828 | 32748 | 4 | 4.5 |
| Sub-group1  (6 strains) | unique | 4733 | 4199 | 18 | 16.7 |
|  | shared | 6710 | 6339 | 18 | 16.7 |
| Sub-group 2  (17 strains) | unique | 15682 | 11848 | 12 | 10.8 |
|  | shared | 30641 | 25640 | 10 | 9.5 |
| Sub-group 3  (25 strains) | unique | 11561 | 9968 | 14 | 12.1 |
|  | shared | 28683 | 24154 | 11 | 10.5 |
| Sub-group 4  (5 strains) | unique | 79591 | 67095 | 5 | 6.0 |
|  | shared | 34495 | 24566 | 5 | 5.5 |

**B**

|  | # of genes with a SV | Mean # of SVs per gene | Mean # SV in LD with nearby SV | Mean # of SNPs in LD with SV |
| --- | --- | --- | --- | --- |
| All 53 strains | 11449 | 4.8 | 1.0 | 4.5 |
| Sub-group1  (6 strains) | 1937 | 2.1 | 1.9 | 16.6 |
| Sub-group 2  (17 strains) | 6268 | 2.6 | 2.1 | 10.0 |
| Sub-group 3  (25 strains) | 5725 | 2.5 | 2.1 | 10.9 |
| Sub-group 4  (5 strains) | 10679 | 4.1 | 3.2 | 5.6 |

**Table S4. BTBR-unique SVs.** The gene name, functional consequence, location, type, and size of BTBR-unique SVs that disrupt exons are shown.

| Gene | Consequence | Location | type | size (bp) |
| --- | --- | --- | --- | --- |
| *Zfp987* | coding_sequence | 4:146124773-146124814 | insertion | 84 |
| *Zfp872* | inframe_deletion | 9:22200462-22200602 | deletion | 134 |
| *Scn11a* | coding_sequence | 9:119778919-119778920 | insertion | 190 |
| *Hmmr* | coding_sequence | 11:40710209-40710210 | insertion | 193 |
| *Trgc3* | coding_sequence | 13:19261724-19262719 | deletion | 996 |
| *Gm17087* | transcript_ablation | 17:8560388-8567457 | deletion | 7070 |
| *Abca17* | coding_sequence | 17:24286687-24289823 | deletion | 3137 |
| *Abca17* | coding_sequence | 17:24290168-24295081 | deletion | 4914 |


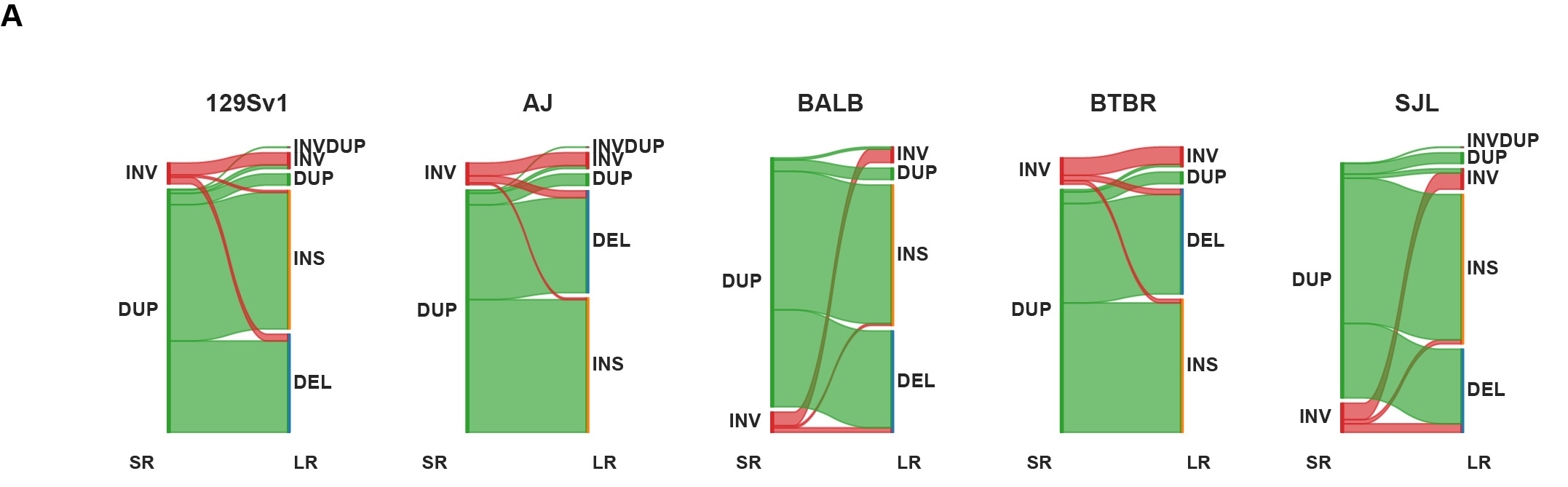


**Figure S1.** Comparison of the type of SV identified by SR and LR sequence analysis. (**A**) These Sankey diagrams indicate the number of SR-SVs that were re-interpreted after analysis of the LR sequence data for each of the 5 inbred strains. Many SR-SV that were identified as inversions or duplications were not confirmed by analysis of the LR sequence data. On average, only 5% of the SV that were identified as duplications by the SR sequence analysis were shown to be not altered after LR sequence analysis, while 61.3% of the inversions identified by SR sequence analysis were found to be not altered after LR sequence analysis.


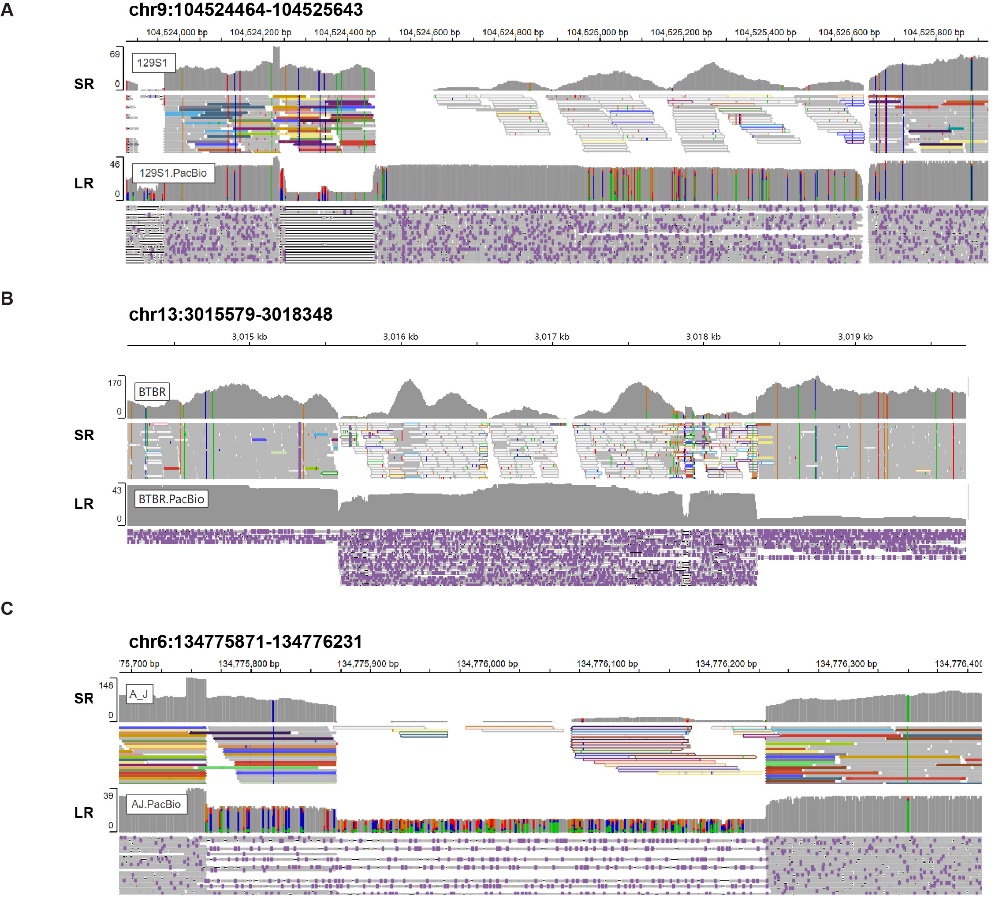


**Figure S2**. Three examples of false positive SR-only homozygous SV calls in the 129S1, BTBR and A/J genomes are shown in panels A-C, respectively. The genomic sequences for regions with the three SVs generated from analysis of SR (top) are shown on top in each panel, and the aligned regions from LR (PacBio) genomic sequence are shown below. While only a few SR sequences aligned to the regions shown in white, these regions are all well covered by the LR sequences.

**Figure S3.** A graph of linkage disequilibrium (LD) decay for 53 inbred mouse strains using alleles that were identified using SNPs alone (black), or those after both SV and SNP alleles were analyzed (red). The half decay distance measured when SNPs and SV alleles were used (30-38 kb) and that obtained when SNP alleles alone were used (31-40 kb) are very similar.


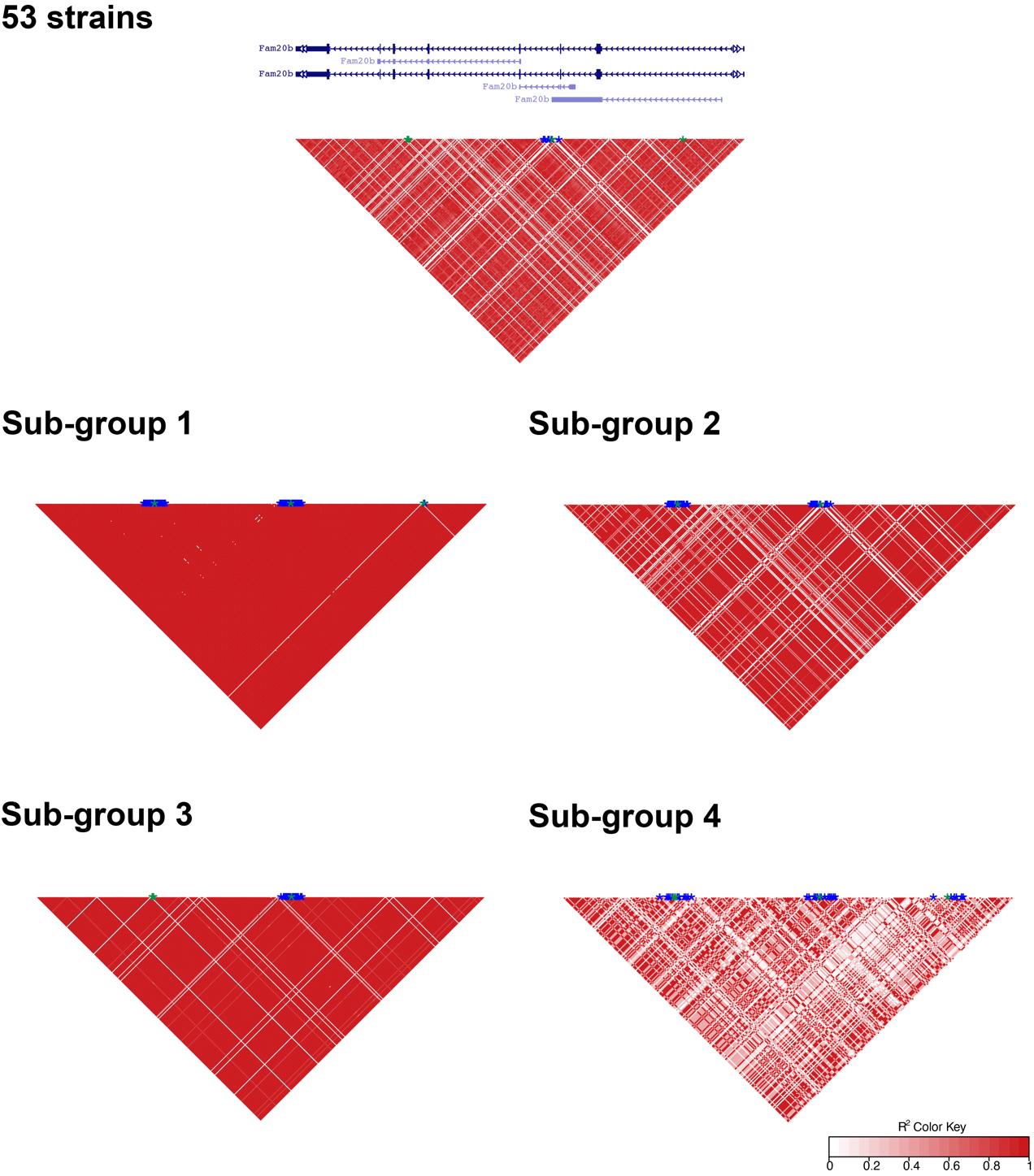


**Figure S4.** LD plots characterizing the relationship between SV and SNP alleles within the 40.5 KB *Fam20b* genomic region. *Top*: This diagram shows the locations of *Fam20b* exons with arrowheads indicating the direction of transcription, which was retrieved from UCSC Genome Browser GRCm38/mm10 mouse assembly. Below the top diagram, the pairwise LD ($r^{2}$) for SVs and SNPs within the *Fam20b* genomic region for the 53-strain panel is shown. Each SV is indicated by a green star. The calculated LD for any pair of allelic variants is indicated by the color of the region within reverse triangle, which is shown in the key below. SNPs that are in the complete LD ($r^{2}=1$) with SVs at nearby sites are indicated by a blue star. *Bottom*: The pairwise LD ($r^{2}$) for SVs and SNPs within the *Fam20b* genomic region for the 4 sub-groups of strains, which were identified by population structure analysis. Thus, *Fam20b* has 4 SVs among the 53-strains, but only one is in complete LD with 6 nearby SNPs. The green stars for the two SVs on the left partially overlap because of their proximity (chr1:156685046 and chr1:156685155), and SNPs that were close to these SVs were not identified. Two SVs are present in the group 2 strains, and they are in complete LD with nearby SNP alleles. One of the 3 SVs present in the group 3 strains is completely linked with 16 nearby SNP alleles among the group 3 strains. In contrast, all 4 of the SVs that are present in the group 4 strains are in complete LD with an average of 7.5 nearby SNPs.


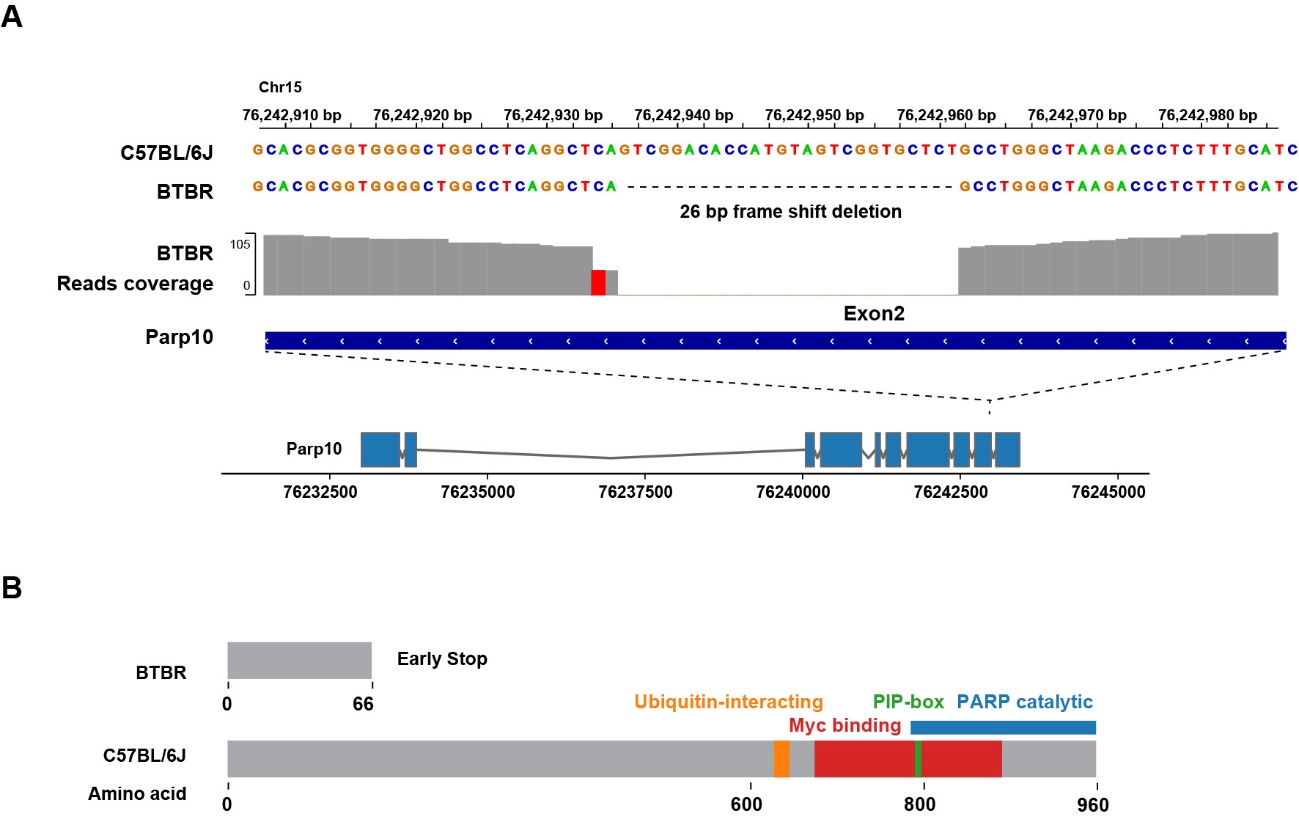


**Figure S5**. BTBR mice produce a non-functional Parp10 protein. **A**) BTBR has a 26 bp deletion within exon 2 of *Parp10* that is not present in 52 other strains with available genomic sequence. **B**) While the full length Parp10 protein has 960 amino acids; the 26 bp frameshift deletion generates a termination codon after amino acid 66. The truncated BTBR protein lacks the catalytic, Myc binding, and ubiquitin-interacting domains that are essential for its role in neurodevelopment.

**
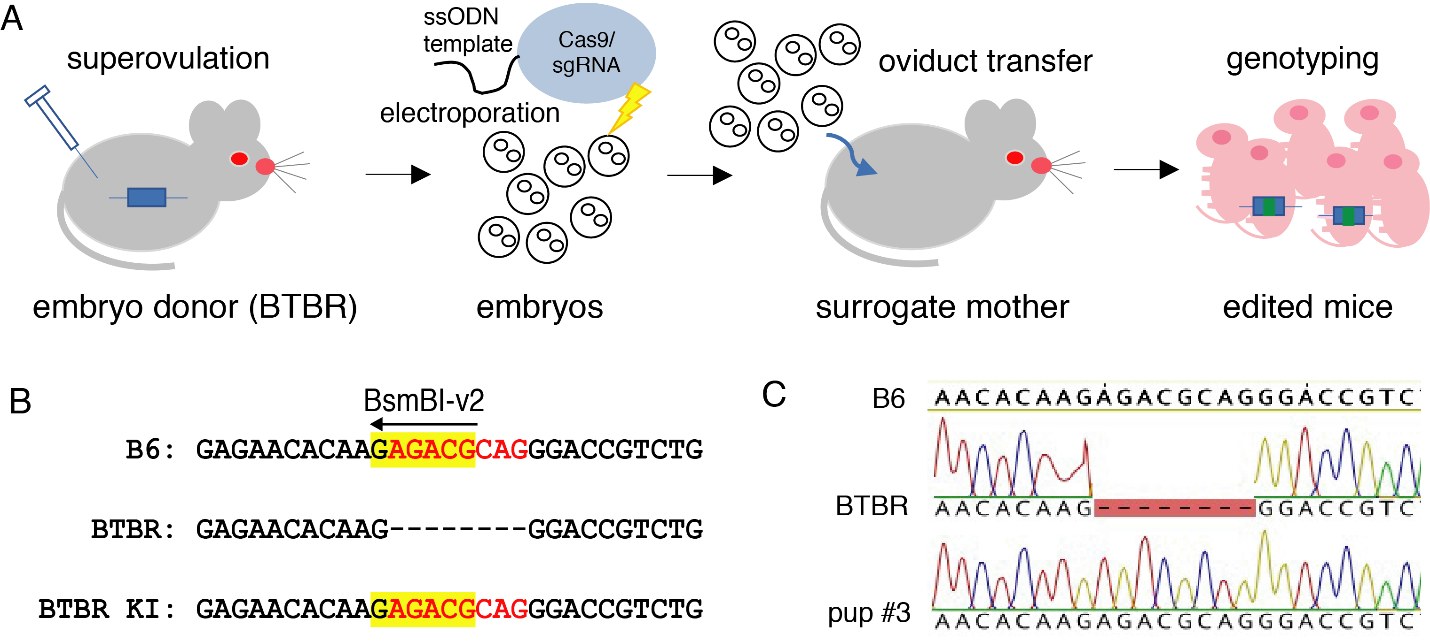
**

**Figure S6**. CRISPR-engineering produces a heterozygous BTBR KI mouse (BTBR *^Draxin WT/-^* KI) with a reversion of the 8 bp deletion in exon 2 of *Draxin* to wild type. (**A**) BTBR female mice were super-ovulated and paired with BTBR males to generate fertilized embryos; and pronucleus (PN) stage embryos were then collected. Cas9, a sgRNA targeting the site in the *Draxin* gene with an 8-bp deletion, and an ssODN with the wild-type sequence were electroporated into embryos. Embryos were transferred into the oviducts of pseudo-pregnant recipients. **(B)** The targeted regions within *Draxin* in C57BL/6, BTBR and BTBR *^Draxin WT/-^* KI mice are shown. The 8-bp (red) sequence in *Draxin* that is present in C57BL/6 mice, which is deleted in BTBR mice, is reverted to wildtype in the BTBR *^Draxin WT/-^* KI mice. The yellow highlighted sequence shows a BsmBI-v2 recognition site that is present in C57BL/6 and BTBR *^Draxin WT/-^* KI mice but is absent in BTBR mice. The BsmBI-v2 recognition site enables pup genomic DNA to be screened for insertion of the 8 bp segment by PCR amplification followed by BsmBI-v2 digestion. **(C)** Genomic DNA obtained from pups was screened by PCR amplification of the targeted region followed by BsmBI-v2 digestion and sequencing. While the 737-bp PCR product from BTBR Draxin is resistant to BsmBI-v2 digestion, the 745-bp PCR product of the BTBR *^Draxin WT/-^* KI mice is cleaved into 440 bp and 305 bp fragments. Sequencing of the digested PCR product from one founder (pup #3) and from a BTBR mouse confirmed that the 8 bp deleted sequence is reverted to wildtype in the BTBR *^Draxin WT/-^* KI. Pup#3 was backcrossed with BTBR to obtain a heterozygous BTBR *^Draxin WT/-^* KI on a clean BTBR background.
